# Usefulness of a cuffless blood pressure monitoring wearable device for aerobic exercise to estimate anaerobic threshold in healthy adults

**DOI:** 10.3389/fphys.2026.1769921

**Published:** 2026-05-20

**Authors:** Taiki Katsumata, Kosuke Shimizu, Naoki Suzuki, Takahiro Miura, Midori Miyagi, Hisako Miura, Wu Xinze, Park Uijin, Koichi Yamamoto, Satoru Ebihara

**Affiliations:** 1 Department of Rehabilitation Medicine, Tohoku University Graduate School of Medicine, Sendai, Japan; 2 Arblet Inc, Tokyo, Japan; 3 Department of Geriatric and General Medicine, the University of Osaka Graduate School of Medicine, Suita, Japan

**Keywords:** aerobic exercise, anaerobic threshold, cardiopulmonary exercise test, cuffless blood pressure monitoring wearable device, double product break point

## Abstract

**Introduction:**

The anaerobic threshold (AT) is an essential indicator of aerobic exercise. However, it is difficult to measure because it requires expensive, spacious equipment and specialized expertise. The double product break point (DPBP) has recently been reported as an alternative indicator of the AT. The purpose of this study was to estimate the usefulness of a cuffless blood pressure monitoring wearable (CBPW) device for determining the AT using the DPBP as an indicator.

**Methods:**

Thirteen healthy adults aged 24–30 years underwent cardiopulmonary exercise testing (CPET), and the oxygen uptake (VO_2_) and heart rate (HR) at the AT as well as at the DPBP (cuff-based sphygmomanometer) were determined. Simultaneously, the CBPW device was worn to measure the DPBP. The agreement between the VO_2_ and HR at the AT, as well as the DPBPs obtained from the cuff-based sphygmomanometer and CBPW device, were evaluated using correlation coefficients and Bland–Altman plots.

**Results:**

Data from the CBPW device enabled the identification of the DPBP in 13 participants. A strong positive correlation was observed between the VO_2_ at the AT and at the DPBP measured by the CBPW device (r = 0.760, p < 0.001), and no fixed or proportional error was found. A strong positive correlation was also found between the HR at the AT and pulse rate at the DPBP measured by the CBPW device (r = 0.945, p < 0.001), but a fixed error with a mean difference of 2.0 (limits of agreement: -7.3 to 11.2) was observed. Correlation was not significant between the VO_2_ at the AT and at the DPBP derived from the sphygmomanometer (r = 0.241, p = 0.476), and between the HR at the AT and at the DPBP derived from the sphygmomanometer (r = 0.217, p = 0.521).

**Conclusion:**

The results indicate that using the CBPW device could enable easier AT estimation without the need for expensive, large equipment, potentially making aerobic exercise more accessible. Conversely, the DPBP measurements obtained with a cuff-based sphygmomanometer showed low agreement with the AT.

## 1 Introduction

Wearable devices and sensors are becoming more readily available to the general population and athletes (Li et al., 2016; Xu et al., 2022). These devices are convenient because they allow for continuous monitoring of patients outside the hospital, customization of treatment (Duking et al., 2020), and simplified workflows for healthcare providers (Haleem et al., 2021). For instance, technological improvements in wearable sensing techniques have made blood pressure measurement using the pulse transit time possible (Mukkamala et al., 2015).

Aerobic exercise promotes the health of the general public and the exercise tolerance of athletes (Arena et al., 2007; Vaitkevicius et al., 1993). Its effectiveness has been well documented in clinical settings for conditions such as hypertension, hyperlipidemia, diabetes, cardiovascular disease, chronic kidney disease, dementia, and chronic obstructive pulmonary disease (COPD) (Cornelissen and Smart, 2013; Smart et al., 2025; Colberg et al., 2016; Battaglia et al., 2024; Avancini et al., 2025; Li et al., 2024). The anaerobic threshold (AT) is defined as the level of work or O2 consumption directly below that at which metabolic acidosis and the associated changes in gas exchange occur (Wasserman et al., 1973), and the heart rate (HR) at the AT is used as an indicator of optimal aerobic exercise (Custodio et al., 2022). Measuring the AT is the gold standard for tailoring exercise intensity to ensure patient safety and therapeutic efficacy and is recommended in major clinical guidelines (Zeng et al., 2023; Patel et al., 2017; Milani et al., 2023). However, AT measurement requires cardiopulmonary exercise testing (CPET), a procedure that necessitates expensive equipment, large spaces, and specialized expertise, resulting in its use in limited facilities.

To overcome these problems, the double product break point (DPBP) has been introduced as an alternative method for estimating the AT. The DPBP is the inflection point of the double product (DP), which is calculated as the product of the HR and systolic blood pressure (SBP). Physiological evidence suggests that the DPBP coincides with metabolic shifts in lactate and catecholamine serum levels (Omiya et al., 2004; Tanaka et al., 1997). For instance, catecholamine surges are known to increase myocardial oxygen consumption (Braunwald, 1971) and are closely associated with the lactate threshold during exercise (Mazzeo and Marshall, 1989).

However, the application of the DPBP is hindered by the limitations of continuous blood pressure measurements. Conventional methods such as arterial lines are highly invasive, and usual blood pressure measurements using cuff-based sphygmomanometers have a low temporal resolution, with measurements typically taken only every 20–30 s, which is insufficient for accurately identifying the break point (Victor de Sousa et al., 2016; Ohtsuki and Watanabe, 2008). There is a noninvasive blood pressure method, such as Finapres (Guelen et al., 2008), which can measure blood pressure beat by beat using a finger cuff, and this method might be considered if we determine the DPBP. However, our new device can measure it cuffless without calibration and a cuff in this small device, and the software. The new device that we developed may enable continuous, non-invasive blood pressure measurements as well as measure the DP. Therefore, it is expected that the DPBP, which is believed to approximate the AT, can be estimated using this device.

The purpose of this study was to investigate whether the DPBP measured using the cuffless blood pressure monitoring wearable (CBPW) device aligns with the AT determined by the gold standard of CPET in healthy adults. Validating this technology is essential for bridging the gap between consumer technology and meaningful medical applications, which could expand access to safe and effective aerobic exercises.

## 2 Materials and methods

### 2.1 Study design

This interventional study was conducted on healthy subjects, approved by the Ethics Committee of the Tohoku University Graduate School of Medicine (approval number: 2023-1-828), and registered in the University Hospital Medical Information Network (UMIN000051911) prior to subject recruitment. The study was conducted between September 2023 and March 2025.

### 2.2 Participants

Eligible participants were healthy adults aged 20–34 years at the time of registration. A power analysis performed with G*Power software (version 3.1.9.7). The sample size for this study was determined based on previous validation studies (Victor de Sousa et al., 2016) demonstrated that the DPBP is a valid estimate of the ventilatory threshold (VT) using subgroups of 9 to 10 participants. Given that the expected correlation between DPBP-derived and CPET-derived thresholds was anticipated to be large (r=0.70) based on these precedents, a sample of 13 participants provides sufficient statistical power (> 0.80) to detect a significant relationship at an alpha level of 0.05. Posters were placed on a bulletin board in Building 1, Seiryo Campus, Tohoku University to recruit participants. The study was explained to the participants and their consent was obtained. The exclusion criteria were presented to all subjects who granted permission, and whether they met each of the requirements was confirmed. The exclusion criteria included individuals who met the absolute contraindications for exercise testing (Liguori and Medicine, 2020), were pregnant or potentially could become pregnant, refused to participate in the study, and deemed inappropriate for study participation by the physician.

### 2.3 Measurement principle of CBPW device

**Figure 1:**
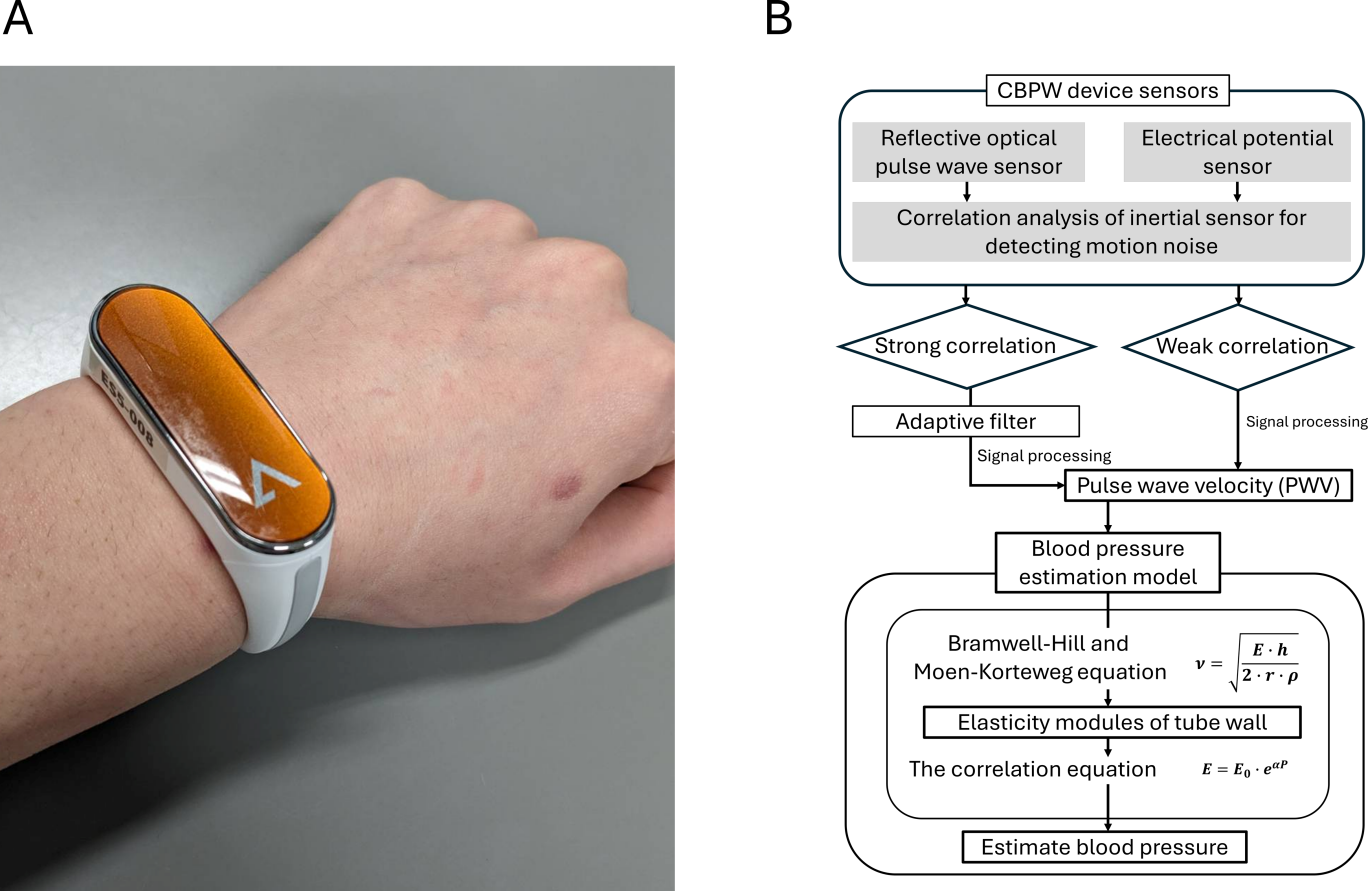
**(A)** Photograph of cuffless blood pressure monitoring wearable (CBPW) device on the wrist. **(B)** Graphical explanation of blood pressure measurement. This diagram illustrates the comprehensive mechanism of the CBPW device for non-invasive blood pressure estimation. It outlines the process from initial sensor data acquisition to final blood pressure estimation, including a robust algorithm for detecting and removing motion noise. V: pulse wave velocity, E: elastic modulus of blood vessel wall, h: vascular wall thickness, r: vascular radius, ρ: blood density, E_0_: zero pressure elastic modulus, a: constant.

We used Alysis Wear manufactured by Arblet Inc.^®^ (Tokyo, Japan) as the CBPW device (**Figure 1A**). This device can simultaneously detect beat-by-beat blood pressure and behavioral information using optical, potential, and inertial sensors. Beat-by-beat blood pressure can be acquired as 10-s.

averaged data. The blood pressure estimation algorithm of this device (Alysis-001) has received medical device approval from the Japanese Ministry of Health, Labour and Welfare (approval number: 30600BZX00212000). Furthermore, an independent clinical validation study comparing this algorithm against 24-hour ambulatory blood pressure monitoring (ABPM) confirmed that it satisfies the rigorous European Society of Hypertension (ESH) awake/asleep test criteria for cuffless blood pressure monitors, demonstrating an average absolute error of ≤5 mmHg and a standard deviation of ≤8 mmHg under resting conditions. To maintain measurement reliability during dynamic activity, the algorithm continuously classifies motion levels using inertial data; blood pressure estimation is designed to automatically suspend during high-intensity exercise when excessive motion artifacts are detected, and resume accurately once the signal stabilizes. This device enables high-frequency DP measurement and the highly accurate, non-invasive derivation of the DPBP. Therefore, we hypothesized that there would be a strong positive correlation and high concordance between the AT measured by CPET and DPBP measured by this device.

Importantly, the device does not acquire a local electrocardiogram (ECG) to derive the pulse transit time. Instead, the device is designed to estimate blood pressure non-invasively and is equipped with two types of sensors: a reflective optical pulse wave sensor and an electrical potential sensor. The reflective optical pulse wave sensor consists of three light wavelengths: green, red, and infrared. This sensor emits light onto the skin surface and the light reflected within the subcutaneous tissue is detected by the light-receiving part. Because hemoglobin in the blood absorbs light of a specific wavelength, it is possible to measure temporal changes in blood volume in the blood vessels accompanying the beating of the heart. As a result, a pulse wave signal based on the blood flow is acquired. Concurrently, electrodes for the skin potential sensor detect subtle bioelectrical signal variations at the skin surface caused by the expansion and contraction of underlying superficial arteries, rather than myocardial depolarization.

The algorithm computes blood pressure continuously using a multi-parametric biomechanical model based on the Bramwell–Hill and Moens–Korteweg equations. Specifically, it integrates three distinct physiological variables extracted from the sensor data (**Figure 1B**):

1. Blood flow speed, which is evaluated by measuring the transit time delay between the peak of the local electro-potential signal and the corresponding peak of the PPG waveform.
2. Changes in arterial volume, which are quantified by analyzing the morphological contour and amplitude of the PPG pulse.
3. Vascular stiffness, which is assessed utilizing the second derivative of the photoplethysmogram (SDPTG) (Park et al., 2021).

Because the SDPTG is widely established in cardiovascular research as a reliable non-invasive indicator of arterial aging and stiffness, dynamically incorporating this index allows the model to precisely track blood pressure fluctuations.By combining the above information on blood velocity, vascular volume, and vascular elasticity, a blood pressure estimation model based on the Bramwell–Hill and Moens–Korteweg equations (Nichols et al., 2011) is constructed in the Arblet cloud installed following the model for detecting blood pressure. This blood pressure estimation model is constructed using the following equation:


$$\nu =\sqrt{\frac{E\cdot h}{2\cdot r\cdot \rho }}$$

v: pulse wave velocity.
E: elastic modulus of blood vessel wall.
h: vascular wall thickness.
r: vascular radius.
ρ: blood density.

Furthermore, the correlation equation between the vascular elasticity coefficient and blood pressure,


$$E=E_{0}\cdot e^{\alpha P},$$

is used to estimate the blood pressure (P).

To address motion-related challenges during exercise, the scaling model of the Alysis-001 algorithm was specifically developed and optimized using diverse datasets that include continuous dynamic movements. Furthermore, this device uses an algorithm consisting of several processes that utilize data from an inertial sensor to extract blood flow information in an environment containing body motion noise associated with exercise, which allows for noise removal. The algorithm is as follows:

Correlation analysis of the inertial and optical sensor data is performed to determine whether the signals obtained from the optical and potential sensors are affected by body motion noise. If the correlation is weak, the effect of body motion noise is assumed to be minimal, and the existing blood pressure estimation algorithm is applied. If the correlation is strong and the impact of body motion noise is clearly observed, the body motion noise is removed.

An adaptive filter is used to remove body motion noise and extract the pulse wave components during exercise. To verify whether the output result of the adaptive filter accurately extracts the pulse wave signal sequentially, logic is constructed to check the continuity with the pulse wave signal captured without filter processing. The pulse wave signal is divided into fixed time-window frames (10 s) and the pulse rates obtained within each window frame interval are compared. This logic analyzes the periodicity and waveform characteristics of the pulse wave signal and detects abnormal values to determine whether the filter output is reliable.

In this comparison, if the pulse wave signal in each window frame varies with continuity, the filter output is adopted as a reliable signal. In contrast, if extreme discontinuity is observed between window frames, the pulse wave signal in the corresponding interval is likely to have been extracted erroneously and is rejected, and no blood pressure value is output.

In the adaptive filter used to remove body motion noise, the body motion data obtained from the inertial sensor are first used as the input signal, and the mixed signal of the body motion noise and pulse wave obtained from the optical sensor is used as the target signal.

A model simulating the body motion noise is then constructed based on the input signal, which is designed to estimate the noise components in the target signal. The filter output generated by the input signal is subtracted from the target signal to obtain the error signal. This error signal contains pure pulse wave components from which the body motion noise has been removed. Analysis of the error signal is used to evaluate whether the filter is operating correctly, and the filter coefficients are successively updated to improve the noise estimation accuracy. This enables the extraction of pulse wave components in dynamic environments.

### 2.4 CPET

CPET was performed once on a treadmill for all subjects. The protocol was based on the guidelines of the American College of Sports Medicine (American College of Sports Medicine et al., 2021), American Heart Association (Fletcher et al., 2013), and treadmill ramp protocol (Yamamoto et al., 1992). The CPET was performed 1–3 h after a meal, and caffeine intake was avoided until 3 h before the meal. The CPET was conducted in a quiet and constant environment (22–24 °C, 40%–60% humidity).

**Figure 2:**
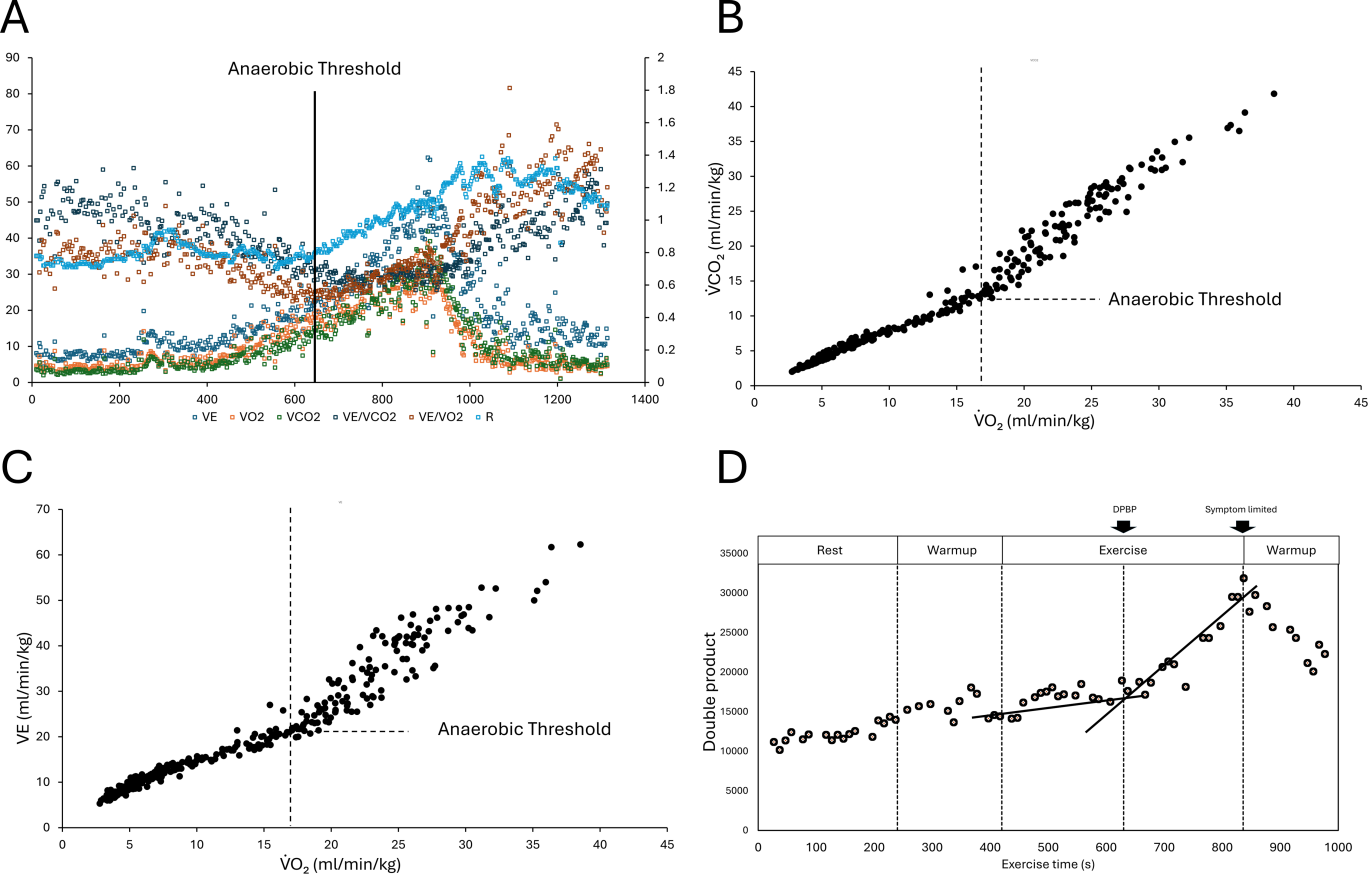
**(A)** Representative example of Anaerobic Threshold (AT) determination during progressive exercise. The vertical black line marks the identified AT. The AT was determined through an integrated assessment of gas exchange parameters, including the ventilatory threshold (VT) and the gas exchange threshold (GET). Key indices shown include minute ventilation (VE), oxygen uptake (VO2), carbon dioxide output (VCO2), ventilatory equivalents for oxygen and carbon dioxide (VE/VO2 and VE/VCO2), and the respiratory exchange ratio (R). The AT is characterized by the point at which VE/VO2 begins to increase without a concurrent rise in VE/VCO2, reflecting the onset of compensatory hyperventilation relative to oxygen consumption. **(B)** Determination of the Anaerobic Threshold (AT) using the V-slope method. The figure shows the relationship between oxygen uptake (VO2) and carbon dioxide output (VCO2) during the cardiopulmonary exercise test. The AT (specifically the gas exchange threshold) was identified as the inflection point (dashed lines) at which the rate of increase in VCO2 relative to VO2 becomes non-linear, typically reflecting the onset of lactic acidosis and subsequent bicarbonate buffering. Data points represent individual breath-by-breath measurements normalized to body weight (ml/min/kg). **(C)** Relationship between minute ventilation (VE) and oxygen uptake (VO2) for AT determination. The graph illustrates the ventilatory response to increasing exercise intensity. The Anaerobic Threshold (AT), or ventilatory threshold (VT), is identified as the point of hyperpnea (dashed lines), where VE begins to increase disproportionately relative to VO2. This shift indicates the onset of compensatory ventilation in response to exercise-induced metabolic acidosis. Each data point represents a breath-by-breath measurement normalized to body weight (ml/min/kg). **(D)** Typical response of the double product (DP) during cardiopulmonary exercise testing (CPET) and double product break point (DPBP). The black lines represent the regression lines for each plot, centered around the inflection point.

AT determination with expiratory gas analysis was conducted by medical doctors and physiotherapists involved in the CPET. Following the criteria adopted by Wasserman et al., and Gaskill et al (Wasserman et al., 1973; Gaskill et al., 2001). The AT was determined using the ventilatory equivalent and V-slope methods (Gaskill et al., 2001). Expired gas flows were measured using a breath-by-breath automated system (Aeromonitor, MINATO Medical Science CO., LTD., Osaka, Japan). Respiratory gas exchange, including ventilation (VE), oxygen uptake (VO2), and carbon dioxide production (VCO2), was continuously monitored (**Figures 2A–C**).

HR and blood pressure were measured using the STS-2100 stress test system (Nihon Kohden Corp., Tokyo, Japan), which was equipped with a Tango M2 automated cuff-based sphygmomanometer (SunTech Medical, Inc., Morrisville, NC, USA). Blood pressure was measured every minute with the arm in a relaxation position or while holding the bar. This equipment doesn’t require the calibration we use in advance.

The CBPW device was worn on the left wrist during CPET to measure blood pressure and pulse rate (PR) with each heartbeat.

### 2.5 DPBP identification method

The data measured by this device were analyzed in the cloud of Arblet Inc. The calculated blood pressure and PR data were obtained as CSV files from Arblet Inc., and the DPBP was identified as described in a previous study (Riley et al., 1997). When identifying the DPBP from the CPET data, we also identified the DPBP from the blood pressure measured every minute using a sphygmomanometer and the HR data from the ECG.

To identify the DPBP, we calculated regression lines for possible combinations using two sets of plots obtained by splitting each plot into two parts. Each set contained at least five plots, and plots from the split sections were included in the regression equation. We then computed the residual sum of squares for each regression line on each plot and identified the intersection point of the two regression lines with the smallest residual sum of squares as the initial DPBP candidate.

To ensure the physiological validity of the identified points, two independent, experienced observers, blinded to the AT results, performed a visual inspection of the calculated DPBP. In cases where the intersection fell outside the data range or when a discrepancy occurred between the algorithm and the observers’ assessment, the DPBP was considered indistinguishable. Any disagreements between the two observers were resolved through adjudication by a third senior evaluator to reach a final decision (**Figure 2D**).

### 2.6 Statistical analysis

All data are expressed as mean ± standard deviation. The correlations were analyzed using Pearson’s product-moment correlation coefficient if the data were normally distributed and tested using Spearman’s rank correlation coefficient if the data were not normally distributed. The correlation coefficients were expressed as r. For the agreement analysis, the limit of agreement (LOA), described as the mean ± 1.96 × standard deviation, was evaluated using the Bland–Altman method (Bland and Altman, 1986) and expressed as the mean difference (lower LOA, upper LOA). Paired t-tests and regression analyses were used to test for fixed and proportional errors. And for quantitative error metrics, we calculated the mean absolute error (MAE). Each test was two-tailed, with a significance level of p < 0.05, and IBM SPSS Statistics Version 26.0 (IBM, Armonk, NY, USA) was used for the analysis.

## 3 Results

All participants who applied for the study met the inclusion criteria and were enrolled after providing informed consent. CPET was feasible in all enrolled subjects. Among all subjects whose data were sent to the Arblet Inc. cloud for analysis, the data of 13 were analyzed. The demographic characteristics of the analyzed subjects were as follows: age: 26.5 ± 4.1 years (20–34 years), height: 173.0 ± 9.4 cm (159–193 cm), weight: 65.3 ± 7.9 kg (50.0–81.8 kg), and BMI: 21.9 ± 2.5 (17.7–26.1). Among these, the DPBP could be identified in 13 cases, of which four were female. In two of the 13 cases, the DPBP could not be identified using CPET data (**Table 1**).

**Table 1:** Demographic characteristics of participants.

Characteristic	Value
Age, years (mean ± SD)	26.0 ± 3.5
Male, n (%)	9 (69.2%)
Height, cm (mean ± SD)	174.0 ± 9.9
Body weight, kg (mean ± SD)	64.9 ± 8.5
BMI, kg/m^2^ (mean ± SD)	21.4 ± 2.47

Values are expressed as means ± standard deviations. BMI: Body mass index.

### 3.1 Measurement accuracy of CBPW device

**Figure 3:**
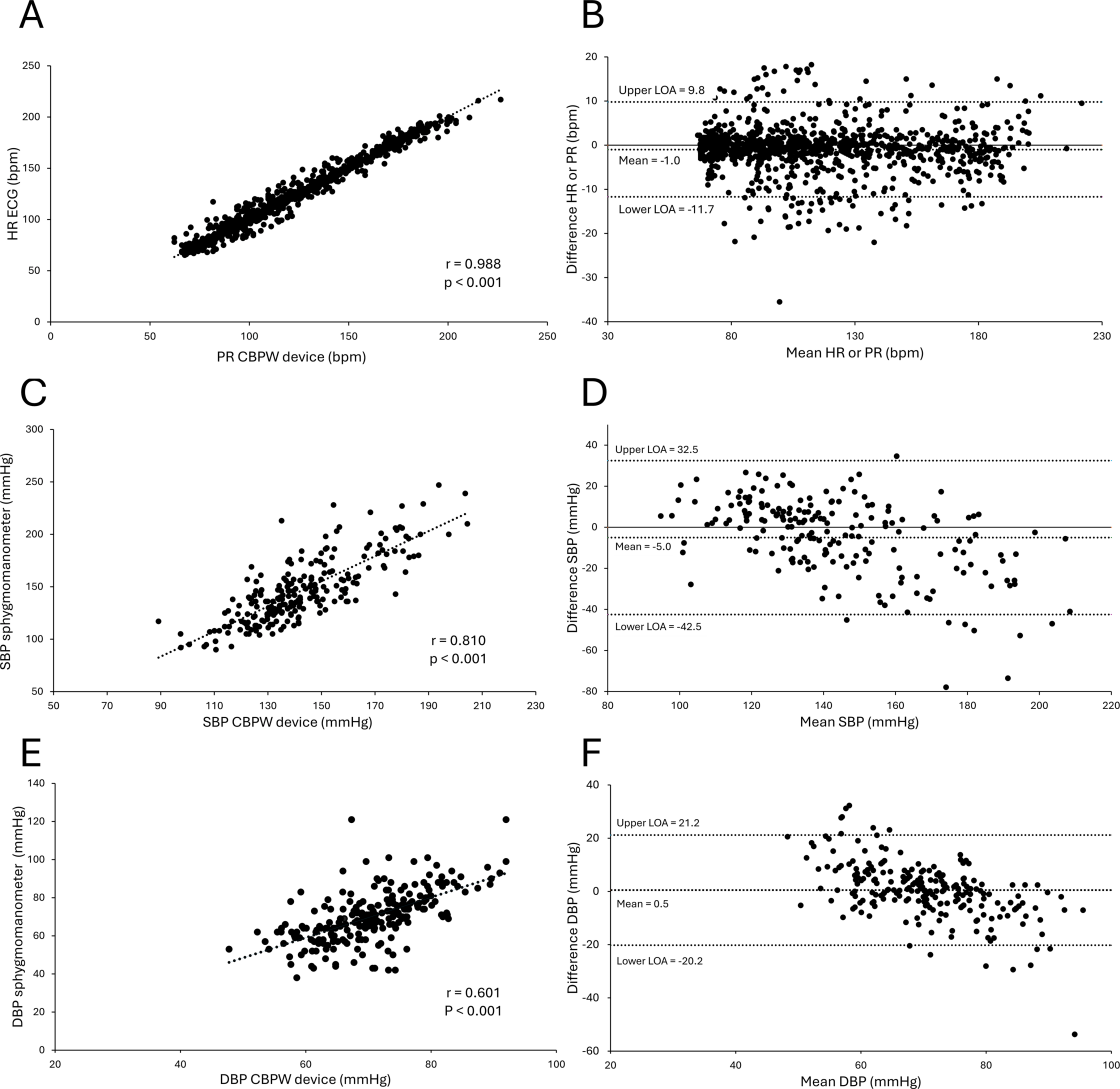
Comparison between the signal data recording device (CBPW device) and the electrocardiogram and cuff-type sphygmomanometer. **(A)** Correlation plot between the pulse rate (PR) measured by the device and heart rate (HR) from electrocardiography (ECG). **(B)** Bland–Altman plot for the PR and HR. **(C)** Correlation plot between the systolic blood pressure (SBP) measured by the CBPW device and a sphygmomanometer. **(D)** Bland–Altman plot for the SBP. **(E)** Correlation plot between the diastolic blood pressure (DBP) measured by the CBPW device and a sphygmomanometer. **(F)** Bland–Altman plot for the DBP.

**Figure 3** shows the correlation and Bland–Altman plots between the CPET and blood pressure, as measured using the CBPW device, and between the HR and PR. The comparison between the HR and PR showed a robust positive correlation of r = 0.988 (p < 0.001) (**Figure 3A**), with a significant difference in the fixed error, but not in the proportional error (**Figure 3B**). A comparison between SBP values showed a strong positive correlation of r = 0.810 (p < 0.001) (**Figure 3C**), whereas both the fixed and proportional errors were significantly different (fixed error: p < 0.001, proportional error: p < 0.001) (**Figure 3D**). A comparison between the diastolic blood pressure (DBP) values showed a positive correlation of r = 0.602 (p < 0.001) (**Figure 3E**), whereas proportional errors were significantly different (p < 0.001) (**Figure 3F**). The mean difference and LOA were -1.0 (-11.7, 9.8) between the HR and PR, -5.0 (-42.5, 32.5) between the SBP values, and 0.5 (-20.2, 21.2) between the DBP values. The indicators measured by the CPET and CBPW device in this study showed strong correlations. MAE each were SBP 14.63 mmHg, DBP 7.48mmHg, HR 3.52 bpm.

### 3.2 Comparison of AT and DPBP using CBPW device

**Figure 4:**
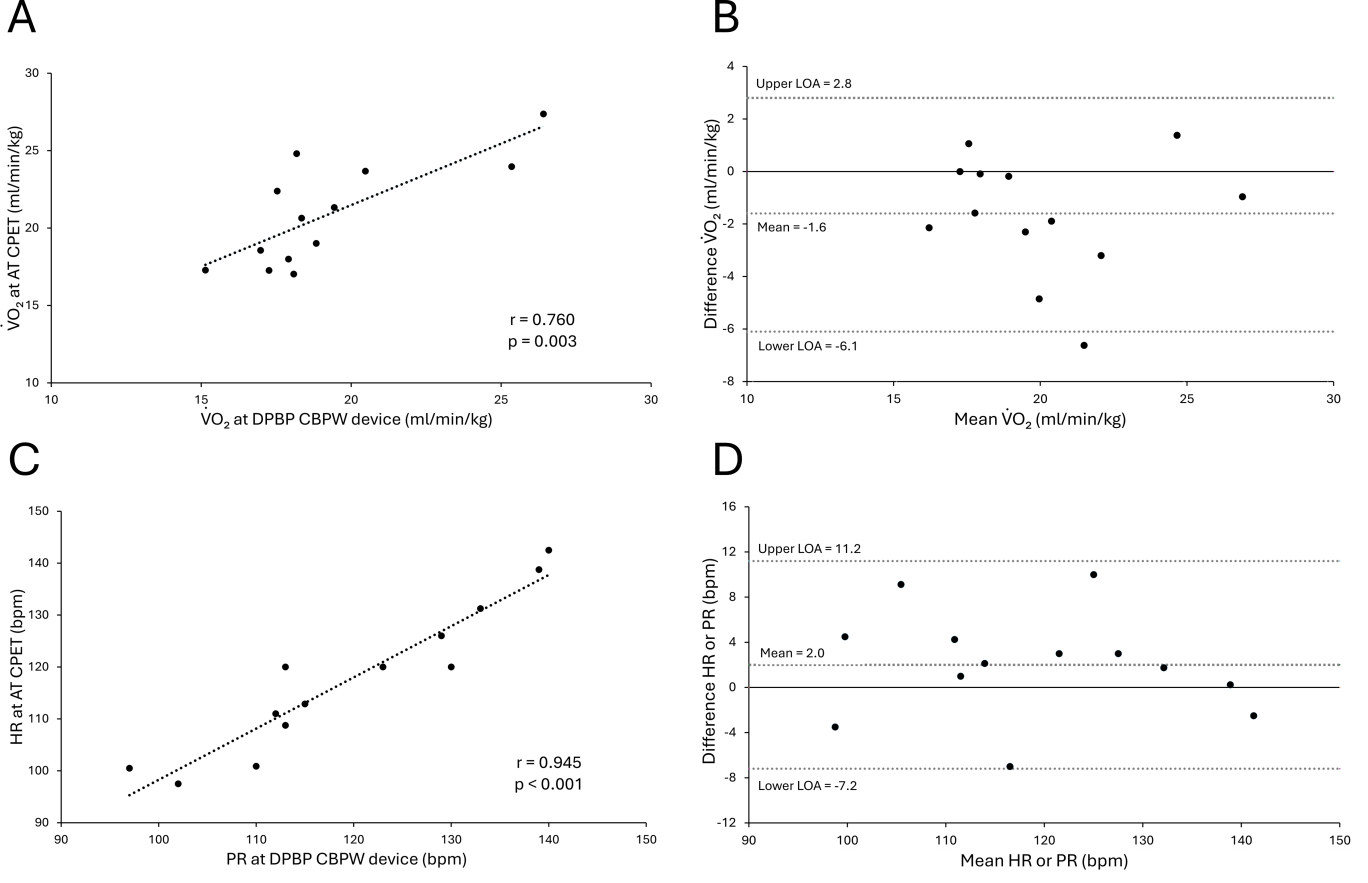
Correlation and agreement between physiological variables at the DPBP identified by the signal data recording device (CBPW device) and the anaerobic threshold (AT) determined by CPET. **(A)** Correlation plot for the oxygen uptake (VO_2_) at the DPBP and AT. **(B)** Bland–Altman plot for the VO_2_ at the DPBP and AT. The y-axis means the reference - device. **(C)** Correlation plot for the PR and HR at the DPBP versus AT. **(D)** Bland–Altman plot for the PR and HR at the DPBP and AT. The y-axis means reference – device.

**Figure 4** shows the correlations and Bland–Altman plots between the VO_2_ at the AT and at the DPBP measured by the CBPW device, as well as between the HR at the AT and PR at the DPBP. The comparison between VO_2_ values showed a strong positive correlation of r = 0.760 (p = 0.003) (**Figure 4A**), with no significant differences in either the fixed or proportional errors (fixed error: p = 0.119, proportional error: p = 0.911) (**Figure 4B**). The comparison between the HR and PR showed a robust positive correlation of r = 0.945 (p < 0.001) (**Figure 4C**), with a significant difference in the fixed error, but not in the proportional error(fixed error: p = 0.001, proportional error: p = 0.350) (**Figure 4D**). The mean difference and LOA were -1.6 (-6.1, 2.8) between VO_2_ values and 2.0 (-7.3, 11.2) between the HR and PR. A very strong positive correlation was observed between the VO_2_ values measured at the AT using CPET and those measured at the DPBP with this device, as well as between the HR at the AT and PR at the DPBP. Additionally, strong agreement was observed in the Bland–Altman plot.

### 3.3 Comparison of AT and DPBP using sphygmomanometer

**Figure 5:**
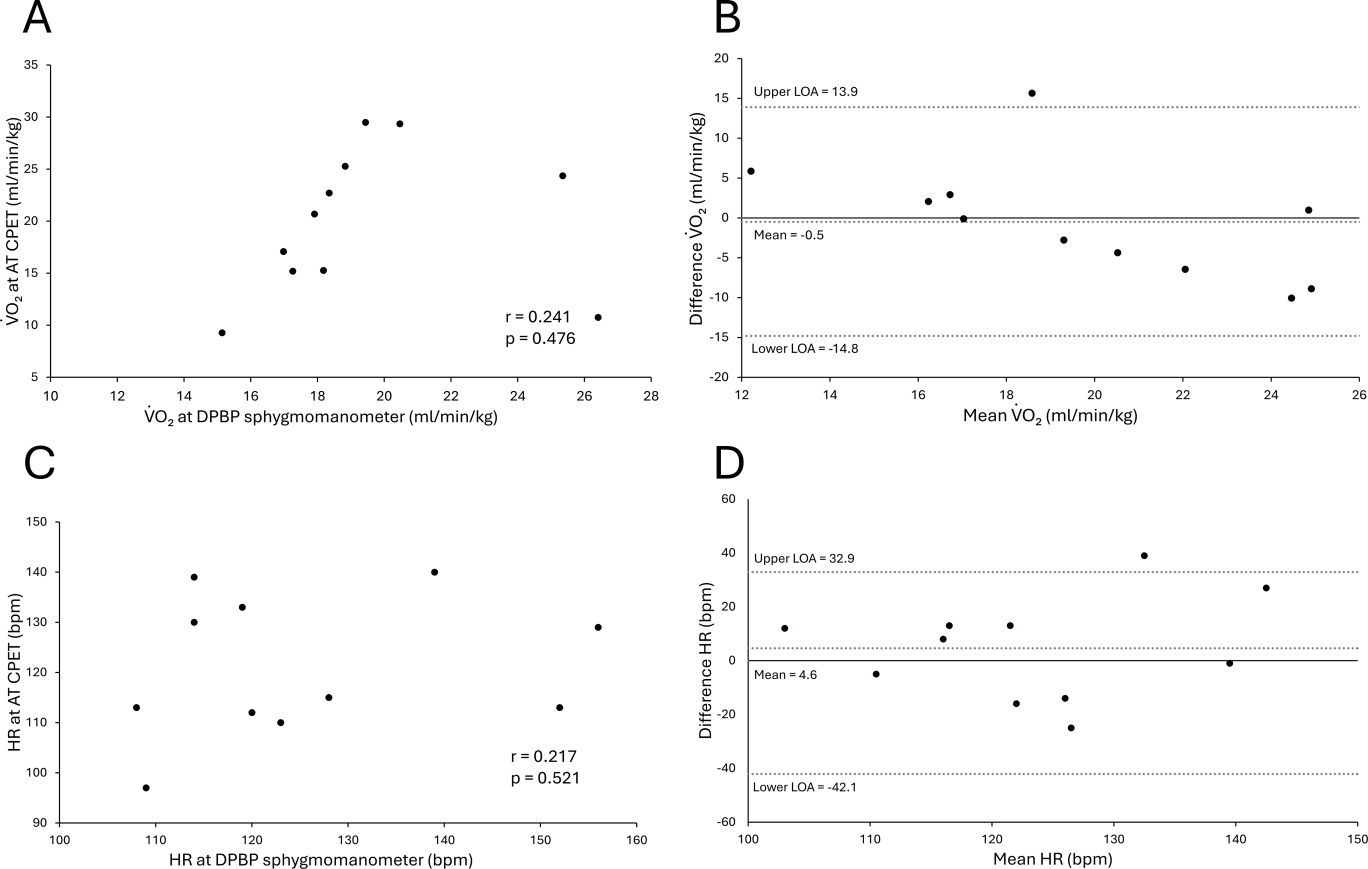
Comparison and agreement between physiological variables at the DPBP identified by a sphygmomanometer and the AT determined by CPET. **(A)** Correlation plot for the VO_2_ at the DPBP and AT. **(B)** Bland–Altman plot for the VO_2_ at the DPBP and AT. The y-axis means reference – device. **(C)** Correlation plot for the PR and HR at the DPBP versus AT. **(D)** Bland–Altman plot for the PR and HR at the DPBP and AT. The y-axis means reference – device.

**Figure 5** shows the correlations and Bland–Altman plots between the VO_2_ at the AT and at the DPBP, as well as between the HR at the AT and at the DPBP, as measured with a sphygmomanometer. In this result, due to incomplete detection for two participants, we show 11 data points. The comparison between VO_2_ values showed no correlation of r = 0.241 (p = 0.476) (**Figure 5A**), with a significant difference in the proportional error, but not in the fixed error (**Figure 5B**). The comparison between the HRs showed no correlation of r = 0.217 (p = 0.521) (**Figure 5C**), with no significant differences in either the fixed or proportional errors (**Figure 5D**). The mean difference and LOA were -0.5 (-14.8, 13.9) between VO_2_ values and -4.6 (-42.1, 32.9) between HR. The correlation coefficient between the VO_2_ and HR at the DPBP obtained from the cuff-type sphygmomanometer during CPET was lower compared with that of the device, and the LOA was also wider than that of the device. The CBPW device could estimate the AT with higher accuracy than the sphygmomanometer during CPET.

## 4 Discussion

The strong positive correlation and acceptable LOAs observed between the VO_2_ values at the AT, as determined by standard CPET, and VO_2_ values at the DPBP, measured by the CBPW device (**Figure 4**), indicate a promising level of consistency. Similarly, the strong correlation between the HR at the AT and PR at the DPBP, as measured by the device, further supports this finding. This is particularly noteworthy considering the capability of the device to monitor the blood pressure and PR beat by beat, which offers a higher temporal resolution than traditional intermittent cuff-based measurements during CPET.

As shown in **Figure 3**, the wide limits of agreement between the CBPW device and the cuff-based sphygmomanometer for SBP during exercise warrant careful consideration. We believe this variance is primarily attributable to a temporal resolution mismatch and asymmetrical limb conditions. Comparing a 60-second intermittent mechanical snapshot with near-continuous, 10-second averaged data during a rapidly progressive ramp exercise naturally introduces temporal discrepancies. Furthermore, the potential influence of differential upper limb movements, with the right arm (cuff) swinging freely and the left arm (CBPW device) being relatively fixed, likely contributed to this variability. Muscle contractions in the swinging arm may have introduced artifacts into the cuff-based measurements, whereas the CBPW device provided stable measurements by motion noise reduction algorithm and placement on a less actively moving limb.

Despite the variance in absolute values, the downstream DPBP analysis remains highly plausible. The primary mathematical objective of DPBP analysis is to detect a relative trend inflection point at submaximal intensity, rather than determining absolute peak blood pressure. Identifying the intersection of two regression lines requires high-density continuous data points. While traditional intermittent cuff-based methods lack the temporal resolution to accurately plot this transition, the near-continuous monitoring capability of the CBPW device inherently provides the necessary data density to capture this specific physiological shift.

The fixed error identified between the HR from the ECG and PR measured using the CBPW device suggests a systematic difference in the underlying physiological signals being captured. Although both reflect cardiac activity, the ECG measures electrical depolarization, whereas the pulse wave sensor detects changes in the peripheral blood volume. This temporal and physiological distinction may account for the observed differences. Despite this fixed error, the strong correlation between the HR and PR indicates that the CBPW device accurately tracks changes in cardiac frequency, which is crucial for DPBP determination. However, this systematic difference should be acknowledged and potentially accounted for when translating DPBP-derived exercise prescriptions based on measured data into those traditionally based on the ECG-derived HR in clinical practice.

Previous studies have reported that the DPBP was observed in patients with diabetes and in cardiac patients taking β-blockers (Kumagai et al., 2002; Vukovich et al., 1979). In terms of exercise therapy, many diseases should be considered, including chronic kidney disease, hypertension, hyperlipidaemia, COPD, cancer, and cardiac disease, for which exercise at the AT level is recommended.

As the DPBP versatility of this device may be expanded in the future by studying not only patients with cardiac disease, but also patients with any other disease, it is necessary to collect knowledge on this device and study its practicality and accuracy for the future replacement of CPET.

The lower correlation and wider LOAs observed with the traditional method likely resulted from the lower sampling frequency of the cuff-based measurements. The ability of the CBPW device to provide near-continuous blood pressure and PR data enables more accurate identification of the DPBP, which appears to reflect the physiological transition at the AT more effectively. This indicates that wearable technology with high-frequency monitoring could offer a more accurate and reliable non-invasive method for estimating the AT than traditional intermittent methods.

This study has several limitations. First, the sample size was relatively small (n = 13 in the DPBP analysis); therefore, interpretations should be made cautiously. In the results of the significance of VO_2_ at AT and DPBP, there might be an influence from the two upper-right portions of the distribution. Although this possibility cannot be ruled out, it could be addressed by increasing the sample size in future work. Second, because the study focused on healthy adults, it is difficult to generalize these results to other patient populations, especially those with cardiovascular disease, arrhythmias, and peripheral vascular disease. These conditions may influence the accuracy of the peripheral pulse wave-based measurements. Third, during the CPET, one arm was maintained stationary to accommodate the CBPW device. However, this setup may not fully reflect typical real-world exercise scenarios in which the arm moves freely. Fourth, although a significant proportional error was observed in CBPW-derived blood pressure compared with the traditional sphygmomanometer, this did not affect the detection of the DPBP inflection point. The strong correlation between DPBP and CPET-derived AT supports the validity of this method for exercise prescription, despite the discrepancy in absolute pressure values. Finally, absolute blood pressure accuracy during high-intensity exercise has not been definitively validated. The device relies on a scaling model to infer blood pressure rather than measuring it directly. While prior studies validating DPBP often utilized direct continuous methods like invasive arterial lines, employing such invasive catheterization during a maximal treadmill ramp test in healthy adults poses prohibitive practical and safety constraints due to intense body movements. Because establishing absolute accuracy during peak dynamic exercise via an arterial line is logistically unfeasible, this study appropriately focused on the device’s ability to capture continuous *relative trends* necessary to mathematically detect the inflection point.

Despite these limitations, this study offers a novel contribution by demonstrating the potential of a cuffless wearable device for non-invasive AT estimation through DPBP monitoring. The findings indicate that the CBPW device could serve as a more accessible and potentially more accurate alternative to traditional CPET for AT assessment, especially in settings with limited specialized equipment and expertise. Future research should aim to validate these results across diverse patient populations, investigate the effects of different exercise modalities and arm movements, and compare the accuracy of the device with invasive blood pressure monitoring to establish its clinical utility in rehabilitation and other areas further.

## 5 Conclusion

This study demonstrated a strong positive correlation and acceptable concordance between the AT measured by CPET and DPBP measured by the CBPW device in healthy adults. Conversely, the DPBP derived from traditional intermittent cuff-based sphygmomanometer measurements showed a weak correlation and poor agreement with the AT. These results suggest that this technology shows promise as a non-invasive alternative to AT estimation, potentially enabling more convenient aerobic exercise. Further research is necessary to validate these findings in clinical populations and characterize the accuracy and reliability of the device across various daily activity conditions.

## Data Availability

The raw data supporting the conclusions of this article will be made available by the authors, without undue reservation.
